# Comparison of Pregnancy and Birth Outcomes Before vs During the COVID-19 Pandemic

**DOI:** 10.1001/jamanetworkopen.2022.26531

**Published:** 2022-08-12

**Authors:** Rose L. Molina, Thomas C. Tsai, Dannie Dai, Mark Soto, Ning Rosenthal, E. John Orav, Jose F. Figueroa

**Affiliations:** 1 Department of Obstetrics and Gynecology, Beth Israel Deaconess Medical Center, Boston, Massachusetts; 2 Harvard Medical School, Boston, Massachusetts; 3 Department of Surgery, Brigham and Women’s Hospital, Boston, Massachusetts; 4 Center for Surgery and Public Health, Boston, Massachusetts; 5 Department of Health Policy and Management, Harvard T.H. Chan School of Public Health, Boston, Massachusetts; 6 PINC AI Applied Sciences, Premier Inc, Charlotte, North Carolina; 7 Division of General Internal Medicine and Primary Care, Brigham and Women’s Hospital, Boston, Massachusetts

## Abstract

**Importance:**

Little is known about changes in obstetric outcomes during the COVID-19 pandemic.

**Objective:**

To assess whether obstetric outcomes and pregnancy-related complications changed during the COVID-19 pandemic.

**Design, Setting, and Participants:**

This retrospective cohort study included pregnant patients receiving care at 463 US hospitals whose information appeared in the PINC AI Healthcare Database. The relative differences in birth outcomes, pregnancy-related complications, and length of stay (LOS) during the pandemic period (March 1, 2020, to April 31, 2021) were compared with the prepandemic period (January 1, 2019, to February 28, 2020) using logistic and Poisson models, adjusting for patients’ characteristics, and comorbidities and with month and hospital fixed effects.

**Exposures:**

COVID-19 pandemic period.

**Main Outcomes and Measures:**

The 3 primary outcomes were the relative change in preterm vs term births, mortality outcomes, and mode of delivery. Secondary outcomes included the relative change in pregnancy-related complications and LOS.

**Results:**

There were 849 544 and 805 324 pregnant patients in the prepandemic and COVID-19 pandemic periods, respectively, and there were no significant differences in patient characteristics between periods, including age (≥35 years: 153 606 [18.1%] vs 148 274 [18.4%]), race and ethnicity (eg, Hispanic patients: 145 475 [47.1%] vs 143 905 [17.9%]; White patients: 456 014 [53.7%] vs 433 668 [53.9%]), insurance type (Medicaid: 366 233 [43.1%] vs 346 331 [43.0%]), and comorbidities (all standardized mean differences <0.10). There was a 5.2% decrease in live births during the pandemic. Maternal death during delivery hospitalization increased from 5.17 to 8.69 deaths per 100 000 pregnant patients (odds ratio [OR], 1.75; 95% CI, 1.19-2.58). There were minimal changes in mode of delivery (vaginal: OR, 1.01; 95% CI, 0.996-1.02; primary cesarean: OR, 1.02; 95% CI, 1.01-1.04; vaginal birth after cesarean: OR, 0.98; 95% CI, 0.95-1.00; repeated cesarean: OR, 0.96; 95% CI, 0.95-0.97). LOS during delivery hospitalization decreased by 7% (rate ratio, 0.931; 95% CI, 0.928-0.933). Lastly, the adjusted odds of gestational hypertension (OR, 1.08; 95% CI, 1.06-1.11), obstetric hemorrhage (OR, 1.07; 95% CI, 1.04-1.10), preeclampsia (OR, 1.04; 95% CI, 1.02-1.06), and preexisting chronic hypertension (OR, 1.06; 95% CI, 1.03-1.09) increased. No significant changes in preexisting racial and ethnic disparities were observed.

**Conclusions and Relevance:**

During the COVID-19 pandemic, there were increased odds of maternal death during delivery hospitalization, cardiovascular disorders, and obstetric hemorrhage. Further efforts are needed to ensure risks potentially associated with the COVID-19 pandemic do not persist beyond the current state of the pandemic.

## Key Points

**Question:**

Was the COVID-19 pandemic associated with changes in pregnancy-related outcomes?

**Findings:**

In a cohort of more than 1.6 million pregnant patients across 463 US hospitals, the number of live births decreased by 5.2% during the COVID-19 pandemic (March 2020 to April 2021) compared with the 14 months prior. While live-birth outcomes and mode of delivery remained stable, small but significant increases in pregnancy-related complications and maternal death during delivery hospitalization were observed.

**Meaning:**

In this study, the COVID-19 pandemic was associated with increases in pregnancy-related complications and maternal deaths during delivery hospitalization.

## Teaser

This cohort study assesses whether obstetric outcomes and pregnancy-related complications changed during the COVID-19 pandemic.

## Introduction

The COVID-19 pandemic has resulted in disruptions to the US health care system, particularly to hospital-based services.^1,2,3,4^ Obstetric care has remained essential to ensure the best outcomes for birthing people and their newborns and has had to rapidly adapt to emerging clinical guidance, patient preferences, and the national spotlight on racial and ethnic inequities in care and outcomes during the pandemic. There are concerns that the pandemic may have negatively affected pregnancy outcomes, as has been shown in other settings around the world.^5,6^ During the pandemic, there has been decreased access to routine in-person outpatient prenatal care and reproductive health services, less monitoring of potential complications, and an increased avoidance of care because of fears of contracting COVID-19.^7^ These concerns are particularly relevant for people with low income and people in minoritized racial and ethnic groups, who experience worse maternal and newborn outcomes at baseline while also facing a disproportionate burden of the COVID-19 pandemic.^8,9,10^ Increased stress from an unprecedented pandemic and related economic crisis may have also led to increases in mental health conditions and worsening physical health for pregnant people.^11,12,13,14,15^ Hospitals have also faced disruptions to medical supply chains, potentially limiting their access to essential supplies for routine obstetric care.^16^

There have been limited empirical studies examining the impact of the COVID-19 pandemic on obstetric outcomes at a national level. Recently, the US Centers for Disease Control and Prevention (CDC) reported concern for increasing maternal mortality in 2018 to 2020; however, this study did not account for changes in risk factors of pregnant people or differences in the underlying quality of hospitals where the births took place and did not examine changes in pregnancy-related complications.^17^ Other work showed no difference in a variety of pregnancy outcomes across 117 health systems.^19^ Birth outcomes have been reported with varied results, with some studies showing decreased preterm birth,^20,21^ others showing no change in preterm birth or stillbirths,^22,23,24,25^ and 1 showing an increase.^18^ Changes to mode of delivery have also been reported with mixed findings.^26,27,28^ Only 1 study^29^ has evaluated incidence of pregnancy complications during the pandemic and found an increase in gestational diabetes, gestational hypertension, poor fetal growth, and preeclampsia, although this study had limited generalizability given that it relied on data from 1 private commercial insurer. Literature on racial and ethnic inequities in obstetric outcomes during the pandemic remains limited.^23,30,31^

Therefore, using a nationwide hospital database, we sought to answer the following key questions. First, were there meaningful changes in maternal death during delivery hospitalization, preterm birth, and mode of delivery during the COVID-19 pandemic compared with the months preceding the pandemic? Second, were there important differences in pregnancy-related complications and delivery hospitalization length of stay (LOS) during the pandemic vs the prepandemic period? Finally, given known preexisting disparities of obstetric outcomes among racial and ethnic minority groups, did the changes over time in obstetric outcomes differ across racial and ethnic groups relative to White patients during the COVID-19 pandemic?

## Methods

### Data Source

The PINC AI Healthcare Database (PHD, previously known as Premier Healthcare Database) is a national hospital-based database containing visit-level information on patient demographic characteristics, diagnoses, and billed procedures. PHD is derived from hospitals on the Premier Quality Advisor Platform that consented for their data to be used for research. It contains more than 1 billion patient encounters and accounts for approximately 25% of all inpatient admissions.^32^ Hospitals in the American Hospital Association (AHA) Annual Survey, compared with those in the PHD, are less frequently located in the South (37.4% vs 43.9%), nonteaching hospitals (59.2% vs 71.7%), rural hospitals (24.1% vs 29.8%), and have greater than 400 beds (10.4% vs 19.6%). Our sample was representative of the overall Premier database (eTable 1 in the Supplement). This data set has been used to identify the association of the COVID-19 pandemic with hospital systems and inpatient mortality.^33,34,35,36^ We identified 463 hospitals where at least 1 birth occurred and that had continuous monthly data from January 1, 2019, to April 30, 2021.

This study was approved by the institutional review board of the Harvard T. H. Chan School of Public Health. Because the PHD contains deidentified data, informed consent of study participants was not pursued. This study followed the Strengthening the Reporting of Observational Studies in Epidemiology (STROBE) reporting guideline.

### Study Variables

There were 3 primary outcomes of this study: the relative change in (1) birth outcomes defined as preterm (<37 weeks’ gestation) vs term; (2) mortality outcomes defined as fetal deaths or stillbirths and maternal death during delivery hospitalization; and (3) mode of delivery defined as vaginal birth, vaginal birth after cesarean (VBAC), primary cesarean birth, repeated cesarean birth, and assisted birth. Mode of delivery was identified using *Current Procedural Terminology *(*CPT*), Diagnosis-Related Group (DRG), and the *International Classification of Disease, Tenth Revision, Procedural Coding System* (*ICD-10-PCS*) (eTable 2 in the Supplement).

Secondary outcomes included the relative change in pregnancy-related complications and delivery hospitalization LOS overall. Ten pregnancy-related complications were assessed: pregnancy-related hypertensive disorders (gestational hypertension, preeclampsia, eclampsia), preexisting hypertensive disorders (chronic hypertension, chronic hypertension with superimposed preeclampsia), cardiovascular or venous conditions (acute myocardial infarction [AMI], cardiomyopathy, venous thromboembolism events [VTEs]), and other obstetric complications (hemorrhage, sepsis). These conditions were identified using *International Classification of Disease, Tenth Revision, Clinical Modification* (*ICD-10-CM*) codes (eTable 2 in the Supplement).

The primary exposure was an indicator of the COVID-19 pandemic period (March 2020 to April 2021) vs the prepandemic period (January 2019 to February 2020). Covariates included the following patient demographic characteristics: age group (<35 years and ≥35 years), insurance (Medicare, Medicaid, commercial, self-pay, and other or unknown), and race and ethnicity, as provided by PHD (Hispanic, non-Hispanic Asian, non-Hispanic Black, non-Hispanic White, and non-Hispanic other/unknown). Race and ethnicity were included as covariates given well-documented disparities in pregnancy and obstetric-related outcomes. We also identified 35 Elixhauser comorbidities, excluding hypertensive disorders and dementia (eTable 3 in the Supplement).^37^

### Statistical Analysis

We compared characteristics of pregnant patients before and during the pandemic using standardized mean differences (SMDs); differences greater than 0.1 were considered meaningfully different. Next, we presented the unadjusted monthly rates of our primary and secondary outcomes across the whole study period.

We used hospital and month fixed-effects regression models to determine the change in each outcome from the prepandemic period to the pandemic period. Logistic regression was used to determine the odds ratio (OR) for binary outcomes and Poisson regression was used to determine the rate ratio (RR) for hospital LOS. Hospital fixed effects were used across all models to avoid confounding by hospital-invariant characteristics. Month fixed effects were used to account for monthly changes in birth patterns.^38^ All models also included the patient covariates listed previously as well as an indicator for preterm (<37 weeks’ gestation) vs term birth. To determine the relative changes in obstetric outcomes between different racial and ethnic groups compared with White patients, we repeated all analyses including an interaction term between race and ethnicity and pandemic period.

In our first sensitivity analysis, we performed a linear regression for LOS (eTable 4 in the Supplement). Second, we replicated our modeling approach but compared outcomes of pregnancies that fully took place during the pandemic (births in January to March 2021) vs pregnancies without exposure to the pandemic (births in January to March of 2019 or 2020) (eTable 5 in the Supplement).

All analyses were conducted in STATA/MP version 16.1 (StataCorp) with 2 -tailed *t* tests, where applicable, and a *P* value of .05 to establish statistical significance. Robust 95% CIs were calculated for all analyses.

## Results

### Patient and Hospital Characteristics

Over the study period there were 1 654 868 birth encounters across 463 hospitals. During the prepandemic period (849 544 births), 153 606 patients (18.1%) were 35 years or older vs 148 274 (18.4%) in the pandemic period (SMD, −0.01). The average gestational age at birth across both periods was 38.3 weeks (SMD, 0.01). The proportion of Asian, Black, Hispanic, and White patients was not meaningfully different before and during the pandemic (eg, Hispanic patients: 145 475 [47.1%] vs 143 905 [17.9%]; SMD, 0.02; White patients: 456 014 [53.7%] vs 433 668 [53.9%]; SMD, 0.00). Differences in insurance (Medicaid: 366 233 [43.1%] vs 346 331 [43.0%]) and comorbidities were also not significant (SMD <0.10). Lastly, 318 pregnant patients (0.04%) were diagnosed with COVID-19 during the pandemic period (Table 1).

**Table 1.** Pregnant Patient Characteristics Before and During the COVID-19 Pandemic, January 2019 to April 2021

Characteristics	Patients, No. (%)		Standardized mean difference
	Pre–COVID-19 period, January 2019 to February 2020 (n = 849 544)	COVID-19 period, March 2020 to April 2021 (n = 805 324)	
Age, y			
<35	695 938 (81.9)	657 050 (81.6)	−0.01
≥35	153 606 (18.1)	148 274 (18.4)	0.01
Term, mean (SD), wk	38.3 (2.42)	38.3 (2.41)	−0.01
Race/ethnicity			
Hispanic	145 475 (17.1)	143 905 (17.9)	0.02
Non-Hispanic			
Asian	37 592 (4.4)	35 629 (4.4)	0.00
White	456 014 (53.7)	433 668 (53.9)	0.00
Black	122 296 (14.4)	116 224 (14.4)	0.00
Other or unknown	88 167 (10.4)	75 898 (9.4)	−0.03
Insurance type			
Commercial	424 669 (50.0)	405 168 (50.3)	0.01
Medicaid	366 233 (43.1)	346 331 (43)	0.00
Self-pay	18 063 (2.1)	14 123 (1.8)	−0.03
Medicare	4930 (0.6)	3922 (0.5)	−0.01
Other	35 649 (4.2)	35 780 (4.4)	0.01
Comorbidities			
Total COVID-19 cases	0	318 (<0.1)	0.03
Deficiency anemias	72 922 (8.6)	79 743 (9.9)	0.05
Chronic blood loss	82 953 (9.8)	87 615 (10.9)	0.04
Coagulopathy	18 716 (2.2)	20 111 (2.5)	0.02
Depression	32 999 (3.9)	38 311 (4.8)	0.04
Diabetes	49 283 (5.8)	54 080 (6.7)	0.04
Substance abuse, drug or alcohol	23 475 (2.8)	24 593 (3.1)	0.02
Hypertension	21 669 (2.6)	22 828 (2.8)	0.02
Chronic pulmonary disease	45 720 (5.4)	49 389 (6.1)	0.03
Obesity	107 869 (12.7)	120 374 (14.9)	0.07

There were 849 544 birth encounters during the prepandemic period. During the pandemic period, the number of birth encounters decreased by 5.2% to 805 324. We found the rates of live births in the PHD were consistent with US census data (eFigure 1 in the Supplement).

### Trends in Mortality Outcomes, Birth Outcomes, Mode of Delivery, and Pregnancy Complications

**Figure 1.**
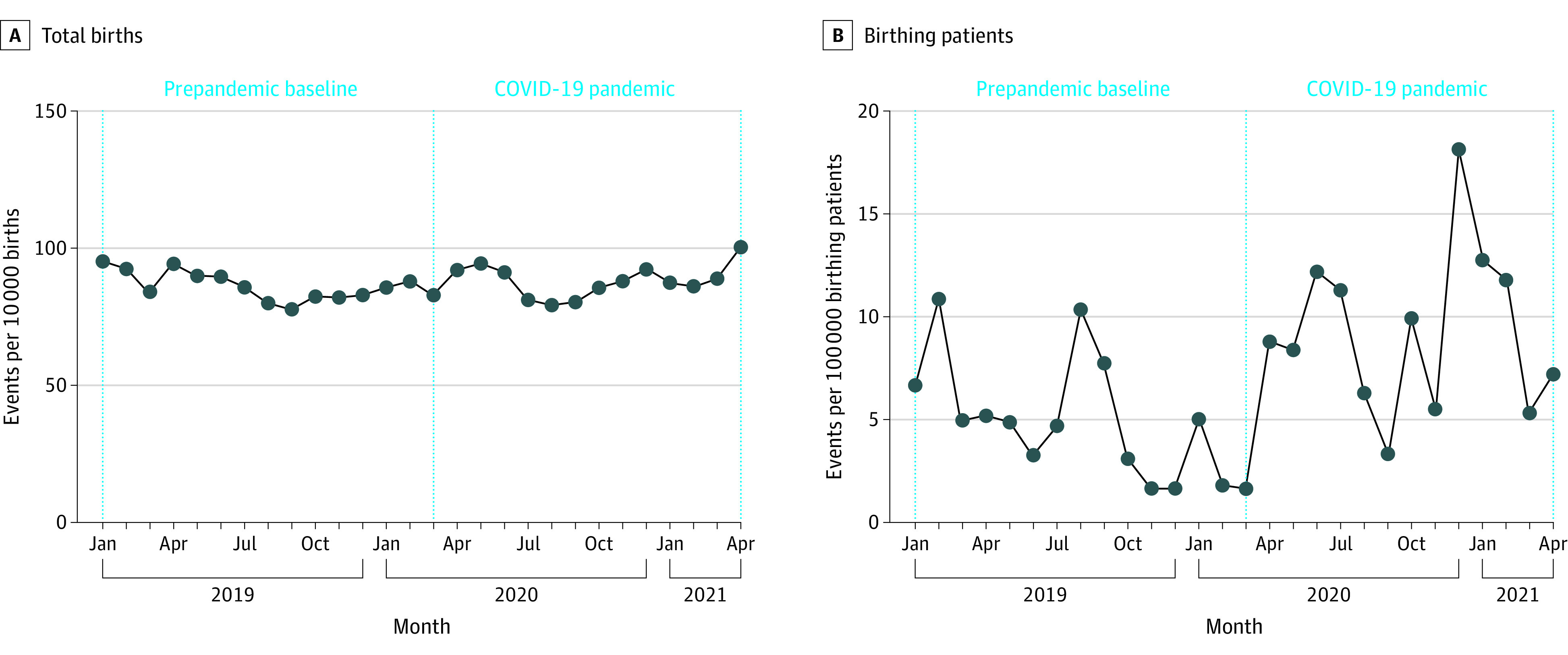
Rates of Mortality Outcomes, January 2019 to April 2021 Vertical dotted line indicates the start of the COVID-19 pandemic.

**Figure 2.**
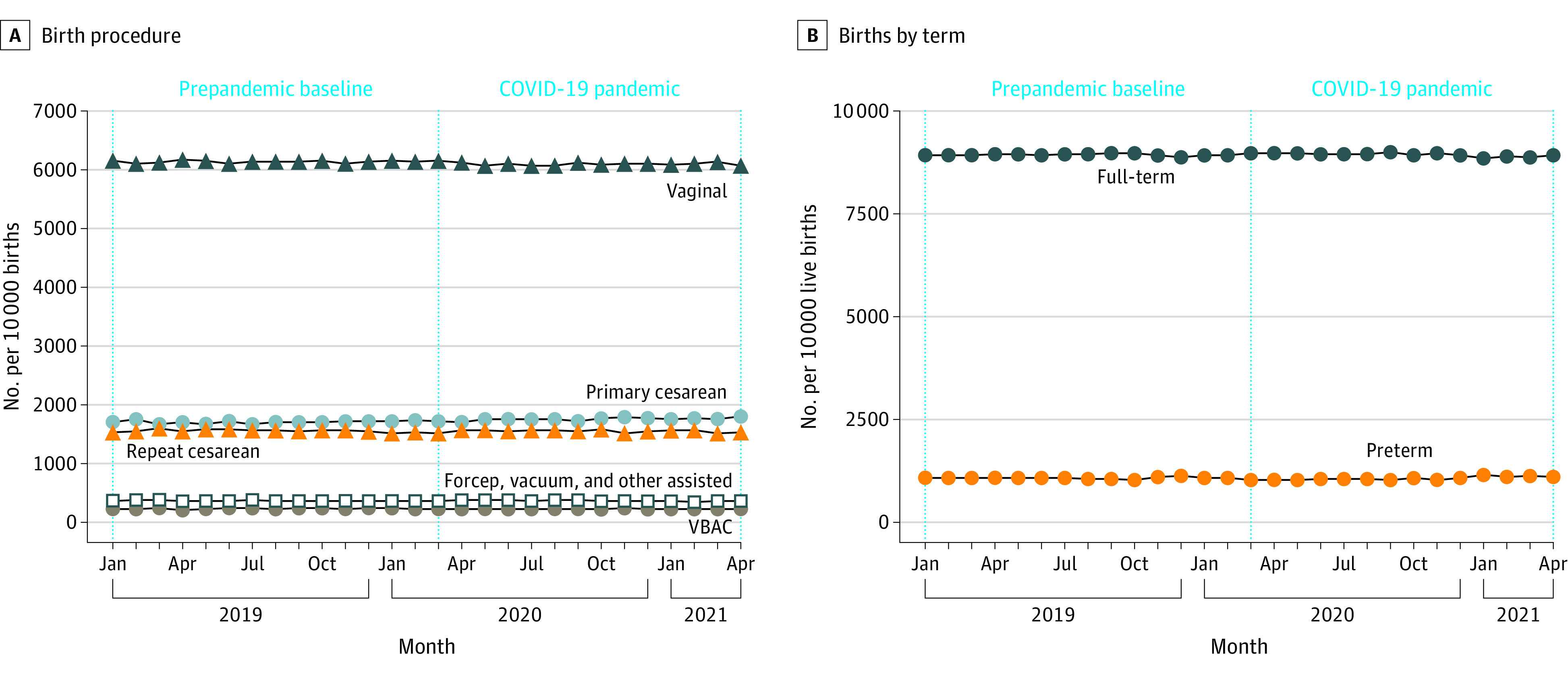
Rates of Live Birth Outcomes and Mode of Delivery, January 2019 to April 2021 Vertical dotted line indicates the start of the COVID-19 pandemic.

Maternal mortality rate during delivery hospitalization was 5.17 per 100 000 pregnant patients in the prepandemic period, which increased to 8.69 per 100 000 pregnant patients during pandemic. The rates of fetal deaths and stillbirths remained relatively stable (prepandemic: 7469 or 0.9% of all births; pandemic: 7196 or 0.9% of all births) (Figure 1; eTable 6 in the Supplement). The rates of preterm and term births remained stable across the study period at 10.7% and 89.3%, respectively (Figure 2B; eTable 4 in the Supplement). Throughout the study period, mode of delivery remained relatively stable (Figure 2B; eTable 6 in the Supplement). Before the pandemic and during the pandemic, 61.5% vs 61.1% of all births were vaginal, 17.0% vs 17.6% were primary cesarean, 15.6% vs 15.5% were repeated cesarean, 3.7% vs 3.7% were assisted births, and 2.3% vs 2.2% were VBAC (eTable 6 in the Supplement).

**Figure 3.**
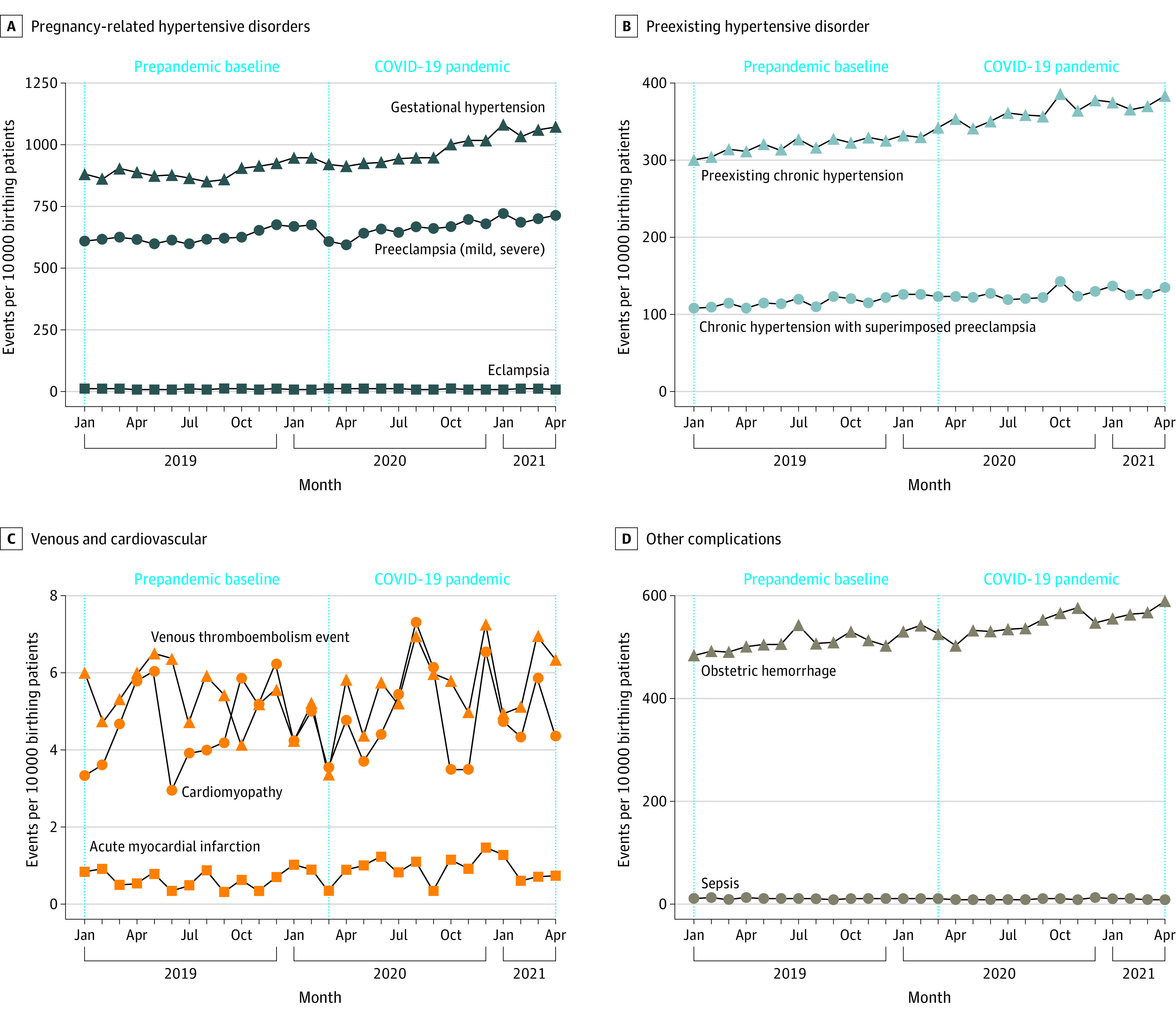
Rates of Complications During Pregnancy and Birth, January 2019 to April 2021 Vertical dotted line indicates the start of the COVID-19 pandemic.

Lastly, the rate of pregnancy complications increased slightly for hypertensive disorders and hemorrhage (Figure 3). Prior to the pandemic, 15.3% of patients had a pregnancy-related hypertensive disorder (8.9% gestational hypertension, 6.3% pre-eclampsia, and 0.1% eclampsia) compared with 16.6% during the pandemic (9.9% gestational hypertension, 6.7% pre-eclampsia, and 0.1% eclampsia). Before the pandemic, 5.1% of patients experienced hemorrhage, compared with 5.5% during the pandemic. Prior to and during the pandemic, the incidence of VTE, sepsis, and cardiomyopathy remained stable at 0.1% (eTable 6 in the Supplement).

### Adjusted Relative Differences During the Pandemic vs Prepandemic Period

The odds of maternal death during delivery hospitalization significantly increased during the pandemic with an adjusted OR of 1.75 (95% CI, 1.19-2.58) (Table 2). There was no significant relative difference in fetal deaths and stillbirths. The odds of primary cesarean delivery underwent a marginal relative increase (OR, 1.02; 95% CI, 1.01-1.04). The odds of having a VBAC did not change (OR, 0.98; 95% CI, 0.95-1.0002), and the odds or having a repeated cesarean birth underwent marginal relative decreases OR, 0.96; 95% CI, 0.95-0.97). The rates of vaginal and assisted births were not significantly different during the pandemic compared with the prepandemic period.

**Table 2.** Relative Differences in Obstetric Outcomes During vs Before the COVID-19 Pandemic

Outcome	OR (95% CI)^a^	*P* value
Mortality outcomes		
Fetal deaths or stillbirths	1.01 (0.98-1.05)	.44
Maternal deaths during delivery hospitalization	1.75 (1.19-2.58)	.004
Mode of delivery		
Vaginal	1.01 (0.996-1.02)	.22
Vaginal birth after cesarean	0.98 (0.95-1.00)^b^	.05
Primary cesarean	1.02 (1.01-1.04)	.005
Repeat cesarean	0.96 (0.95-0.97)	<.001
Forceps, vacuum, and other assisted	1.01 (0.99-1.04)	.40
Complications		
Pre-existing chronic hypertension	1.06 (1.03-1.09)	<.001
Chronic hypertension with superimposed pre-eclampsia	1.01 (0.97-1.05)	.56
Gestational hypertension	1.08 (1.06-1.11)	<.001
Pre-eclampsia (mild, severe)	1.04 (1.02-1.06)	.001
Eclampsia	1.02 (0.91-1.14)	.72
Sepsis	0.89 (0.80-0.99)	.03
Obstetric hemorrhage	1.07 (1.04-1.10)	<.001
Acute myocardial infarction	1.38 (0.94-2.01)	.10
Cardiomyopathy	0.94 (0.79-1.10)	.43
Venous thromboembolism events	0.95 (0.81-1.11)	.50
Length of stay, mean, RR (95% CI)^c^		
All live births	0.931 (0.928-0.933)	<.001
Vaginal	0.94 (0.94-0.94)	<.001
Cesarean	0.91 (0.91-0.92)	<.001
Assisted	0.95 (0.94-0.96)	<.001

Abbreviations: OR, odd ratio; RR, rate ratio.

^a^ Results from a logistic model to assess the relative difference in obstetric outcomes during the COVID-19 pandemic (March 2020 to April 2021) vs before the pandemic (January 2019 to February 2020).

^b^ The upper 95% CI for the OR of vaginal birth after cesarean is 1.0002.

^c^ Results are from a Poisson model to assess the relative difference in length of stay during birth hospitalization during the COVID-19 pandemic (March 2020 to April 2021) vs before the pandemic (January 2019 to February 2020).

Some hypertensive disorders underwent marginal but statistically significant increases during the pandemic (gestational hypertension: OR, 1.08; 95% CI, 1.06-1.11; preeclampsia: OR, 1.04; 95% CI, 1.02-1.06; preexisting chronic hypertension: OR, 1.06; 95% CI, 1.03-1.09). Odds of obstetric hemorrhage also marginally increased (OR 1.07, 95% CI, 1.04-1.10). Odds of sepsis decreased (OR, 0.89; 95% CI, 0.80-0.99) (Table 2). There were no significant changes in other complications.

Lastly, the LOS during delivery hospitalization significantly decreased across all modes of delivery by 7% (RR, 0.931; 95% CI, 0.928 to 0.933). More specifically, the LOS for cesarean, vaginal, and assisted births decreased by 9%, 6%, and 5%, respectively (cesarean: RR, 0.91; 95% CI, 0.91-0.92; vaginal: RR, 0.94; 95% CI, 0.94-0.94; assisted: RR, 0.95; 95% CI, 0.94-0.96) (Table 2).

### Adjusted Differences by Race and Ethnicity

When comparing across race and ethnicity, we observed higher rates of hypertensive disorders among Black patients before and during the pandemic (eFigure 2 in the Supplement). However, there were very few variations across race and ethnicity of the risk-adjusted changes in obstetric outcomes during the pandemic compared with the prepandemic period. For example, there were no significant differences in the change in fetal deaths and stillbirth or maternal deaths during delivery hospitalization that occurred during the pandemic between Black and White patients (stillbirth or fetal deaths: OR, 0.99; CI, 0.90-1.08; maternal death during delivery hospitalization: OR, 0.47; 95% CI, 0.17 to 1.26) (eTable 7 in the Supplement).

### Sensitivity Analysis

In our sensitivity analyses, the directionality of all terms was identical to our main analyses. Details appear in eTables 4 and 5 in the Supplement.

## Discussion

In a national sample across 463 hospitals, we found a small but significant increase in maternal death during delivery hospitalization, hypertensive disorders, and hemorrhage during the pandemic compared with before. Rates of preterm births and mode of delivery remained relatively stable despite a reduction in total live births by 5.2% during the pandemic period. LOS after birth also decreased during the pandemic. There were no racial or ethnic differences in the relative changes in obstetric outcomes in the pandemic period compared with the prepandemic period. Taken together, our study raises concerns that the COVID-19 pandemic may have negatively affected obstetric care and pregnancy-related outcomes.

We observed declines in the number of live births in a hospital setting during the pandemic, which was consistent with Census reports. Much of the drop experienced, however, was between March and December 2020, which mostly reflects pregnancies that began prior to the period of the pandemic. The decision to conceive is sensitive to the social context, and pandemic circumstances may have led to fewer people to expand families. Alternatively, this might be explained by an increase in home births during 2020.^39^ However, our results match the trend reported in Census reports, which also capture home births, so increased home births is unlikely to be a major driver.

Despite the external pandemic stresses on health systems, mode of delivery remained stable. While the pandemic magnified health inequities in other clinical fields, we found that preexisting inequities in maternal and birth outcomes did not worsen during the pandemic. While it is concerning that pregnant Black patients on average had higher rates of hypertensive complications before and during the pandemic, it is reassuring that these underlying inequities in care did not worsen in our data set. However, there are signals of worsening inequities in maternal deaths among non-Hispanic Black birthing people according to a recent report from the CDC.^17^ Our work differs in that it only includes maternal death during delivery hospitalization, while the CDC report reflects deaths from day of pregnancy to 42 days after pregnancy ends; therefore, the CDC findings might reflect higher deaths outside the hospital setting among Black patients.

The increase in maternal death during delivery hospitalization and pregnancy-related complications during the pandemic is alarming. A recent study has shown that SARS-CoV-2 infection during pregnancy is associated with higher risk of death or serious morbidity.^40^ While SARS-CoV-2 was low in our sample, our work extends these findings to demonstrate the associations of the overall disruptions of the COVID-19 pandemic with the health of pregnant people. While hospital-based obstetric care remained an essential service during the COVID-19 pandemic, outpatient prenatal care experienced substantial disruptions, and much routine care was done virtually.^41,42^ It is possible that these disruptions and limitations in monitoring with telehealth may have contributed to the slight worsening of pregnancy-related complications. Additionally, increased rates of hypertensive disorders may be due to heightened stress provoked by the pandemic, or reluctance to engage in for prenatal care due to concerns about COVID-19 exposure. As the nation continues to face ongoing surges, it will be important to mitigate further pandemic-related disruptions on obstetric care and pregnancy outcomes.

The pandemic context also facilitated early postpartum discharge protocols to minimize exposure risks in the hospital and allow for surge capacity.^43^ We found decreased LOS during delivery hospitalization across all modes of delivery, but especially after cesarean. This is consistent with a study^44^ that showed decreased LOS without an increase in adverse maternal or neonatal outcomes. Interestingly, sepsis rates in our sample declined, which is possibly because of enhanced hand hygiene and masking due to the COVID-19 pandemic.

Notably, we did not find that relative changes in obstetric outcomes differed significantly by race and ethnicity, indicating that preexisting inequities among Black patients in obstetric outcomes persisted but did not worsen during the pandemic in this national sample. While obstetric operations mobilized to adapt to rapidly changing clinical guidance and maintain essential services, the experience of care was dramatically different, as noted through restrictive visitation policies,^45,46^ which limited social support^47^ during a particularly anxiety-provoking hospitalization.^48,49^ People of color were more likely to experience disrespectful care^50^ and perceived discrimination and have lower birth satisfaction, which were associated with postpartum stress and birth-related posttraumatic stress disorder.^51^

### Limitations

Our study has several limitations. First, while PHD is broadly representative of US acute care hospitals, it is possible that the results do not reflect hospitals that do not participate in the registry. Second, PHD relies on accurate reporting of obstetric care codes, procedures, and patient demographics. Third, given the observational nature of the study, we cannot definitively conclude causality between our exposure (the COVID-19 pandemic period) with our outcomes of interest. Fourth, because of limitations of the data set, we were unable to verify precise causes of maternal death given that we lacked data from death certificates. We also acknowledge that we were unable to ascertain whether the people who gave birth during the pandemic period had more comorbidities at baseline compared with reproductive-age people who deferred childbearing during this time. Fifth, we have chosen not to include potential mediators in our models to capture the full association of the pandemic; for example, when modeling LOS, we chose not to include birth complications as covariates since those complications could have been the result of the pandemic and their inclusion would have artificially weakened the association with the pandemic. Sixth, we were unable to assess parity in the context of vaginal birth rates since that information is not available in the database; however, we were able to distinguish primary vs repeated cesarean births and VBAC. Additionally, patients who gave birth during the pandemic period may have had varying exposures to the pandemic during their pregnancy. However, our sensitivity analysis comparing patients with full exposure to the pandemic during pregnancy with those without indicated that our findings were robust despite potential variation in pandemic exposure.

## Conclusions

In a large national database of US hospitals, we found that while overall births decreased, mode of delivery and preterm births remained stable. However, we observed small but concerning increases in maternal death during delivery hospitalization, hypertensive disorders, and postpartum hemorrhage during the first 14 months of the COVID-19 pandemic.

## Notes

Supplement. **eTable 1.** Characteristics of Hospitals in the Premier Healthcare Database Compared With the American Hospital Association Annual Survey, 2018 **eTable 2.** *ICD-10*, *CPT*, and DRG Codes for Study Outcomes **eTable 3. **Elixhauser Comorbidities, During COVID-19 vs Pre–COVID-19 **eTable 4. **Sensitivity Analysis: Multivariate Linear Regression for Absolute Difference in Length of Stay by Mode of Delivery, During COVID-19 vs Pre–COVID-19 **eTable 5.** Sensitivity Analysis: Relative Difference in Obstetric Outcomes, Pregnancies That Took Place Fully During the Pandemic vs Those Without Exposure to the Pandemic **eTable 6. **Unadjusted Obstetric Outcomes, During COVID-19 vs Pre–COVID-19 **eTable 7. **Multivariate Logistic Regression for Relative Difference in Obstetric Outcomes Across Race and Ethnicity, During COVID-19 vs Pre–COVID-19 **eFigure 1. **Comparing Live Birth Data From US Census and PHD, 2021 and 2020 vs 2019 **eFigure 2. **Rates of Complications During Pregnancy and Birth by Race and Ethnicity, January 2019 to April 2021
