# Errors in Abstract, Results, and Figure

**DOI:** 10.1001/jamanetworkopen.2022.33824

**Published:** 2022-09-02

In the Original Investigation titled “Comparison of Pregnancy and Birth Outcomes Before vs During the COVID-19 Pandemic,”^1^ published August 12, 2022, there were errors in the Abstract, Results, and Figure 1. In the Abstract and Results, there was a typographical error in the percentage of Hispanic patients in the prepandemic period. It should have read 17.1%. Additionally, the title of panel A in Figure 1 should have read “Fetal death or stillbirth,” and the title of panel B should have read “Maternal death during delivery hospitalization.” This article has been corrected.^1^
