# Errors in Results, Discussion, Table 1, and the Supplement

**DOI:** 10.1001/jamanetworkopen.2023.14781

**Published:** 2023-05-03

In the Original Investigation titled “Comparison of Pregnancy and Birth Outcomes Before vs During the COVID-19 Pandemic,”^1^ published August 12, 2022, there were errors in the Results and Discussion sections as well as in Table 1 and eTable 6 in the Supplement. In Table 1 and the Results section, the number of pregnant patients diagnosed with COVID-19 during the pandemic period should have read 15 461 (1.9%). In Table 1, the standardized mean difference for total COVID-19 cases between the pre–COVID-19 and COVID-19 periods should have read 0.20. In eTable 6 in the Supplement, the number of maternal deaths with COVID-19 diagnosis should have read 22 (0.0%), and the third sentence of the fourth paragraph in the Discussion should have read, “Our work extends these findings to demonstrate the associations of the overall disruptions of the COVID-19 pandemic with the health of pregnant people.” This article has been corrected.^1^
