# Supplementary material for: Comparison of Pregnancy and Birth Outcomes Before vs During the COVID-19 Pandemic
Source: JAMA Netw Open. 2022 Aug 12;5(8):e2226531. doi: 10.1001/jamanetworkopen.2022.26531 (PMC9375166; doi:10.1001/jamanetworkopen.2022.26531)
Supplement: Supplement. — eTable 1. Characteristics of Hospitals in the Premier Healthcare Database Compared With the American Hospital Association Annual Survey, 2018 eTable 2. ICD-10, CPT, and DRG Codes for Study Outcomes eTable 3. Elixhauser Comorbidities, During COVID-19 vs Pre–COVID-19 eTable 4. Sensitivity Analysis: Multivariate Linear Regression for Absolute Difference in Length of Stay by Mode of Delivery, During COVID-19 vs Pre–COVID-19 eTable 5. Sensitivity Analysis: Relative Difference in Obstetric Outcomes, Pregnancies That Took Place Fully During the Pandemic vs Those Without Exposure to the Pandemic eTable 6. Unadjusted Obstetric Outcomes, During COVID-19 vs Pre–COVID-19 eTable 7. Multivariate Logistic Regression for Relative Difference in Obstetric Outcomes Across Race and Ethnicity, During COVID-19 vs Pre–COVID-19 eFigure 1. Comparing Live Birth Data From US Census and PHD, 2021 and 2020 vs 2019 eFigure 2. Rates of Complications During Pregnancy and Birth by Race and Ethnicity, January 2019 to April 2021 [file jamanetwopen-e2226531-s001.pdf]

Supplemental Online Content

Molina RL, Tsai TC, Dai D, et al. Comparison of pregnancy and birth outcomes before vs during the COVID-19 pandemic. *JAMA Netw Open.* 2022;5(8):e2226531. doi:10.1001/jamanetworkopen.2022.26531

eTable 1. Characteristics of Hospitals in the Premier Healthcare Database Compared With the American Hospital Association Annual Survey, 2018

eTable 2. *ICD-10*, *CPT*, and DRG Codes for Study Outcomes

eTable 3. Elixhauser Comorbidities, During COVID-19 vs Pre–COVID-19

eTable 4. Sensitivity Analysis: Multivariate Linear Regression for Absolute Difference in Length of Stay by Mode of Delivery, During COVID-19 vs Pre–COVID-19

eTable 5. Sensitivity Analysis: Relative Difference in Obstetric Outcomes, Pregnancies That Took Place Fully During the Pandemic vs Those Without Exposure to the Pandemic

eTable 6. Unadjusted Obstetric Outcomes, During COVID-19 vs Pre–COVID-19

eTable 7. Multivariate Logistic Regression for Relative Difference in Obstetric Outcomes Across Race and Ethnicity, During COVID-19 vs Pre–COVID-19

eFigure 1. Comparing Live Birth Data From US Census and PHD, 2021 and 2020 vs 2019

eFigure 2. Rates of Complications During Pregnancy and Birth by Race and Ethnicity, January 2019 to April 2021

This supplemental material has been provided by the authors to give readers additional information about their work.

© 2022 Molina RL et al. *JAMA Network Open.*

eTable 1. Characteristics of Hospitals in the Premier Healthcare Database Compared With the American Hospital Association Annual Survey, 2018

	Study Sample 2019-2021	Premier Database 2018	AHA Database 2018
**Number of Hospitals**	463	766	4354
**Size (%)**			
000-199 beds	40.8%	56.5%	71.3%
200-399 beds	35.4%	25.6%	18.4%
400+ beds	23.8%	17.9%	10.3%
**Teaching hospital status (%)**			
Teaching hospital	69.3%	71.7%	59.2%
Non-teaching hospital	30.7%	28.3%	40.8%
**Region (%)**			
Northeast	29.8%	15.0%	12.2%
Midwest	9.5%	26.6%	30.2%
South	43.6%	43.9%	37.4%
West	17.1%	14.5%	20.1%
**Geography (%)**			
Rural	27.6%	29.8%	24.1%
Urban	72.4%	70.2%	75.9%

© 2022 Molina RL et al. *JAMA Network Open.*

eTable 2. *ICD-10*, *CPT*, and DRG Codes for Study Outcomes

**Delivery Mode**	
Vaginal	Live birth with CPT code 59409, 59612, 59400, or 59610, or DRG code 768, 796, 797, 798, 805, 806 or 807
VBAC	Vaginal birth with ICD 10 Code O34.21 or O34.22 (Maternal care for scar or isthmocele from previous cesarean delivery).
Primary Cesarean	Delivery encounter with CPT Code 59510 or 59618 or DRG Code 783, 784, 785, 786, 787, 788 without ICD 10 Code O34.21 or O34.22.
Repeat Cesarean	Cesarean delivery with ICD 10 Code O34.21 or O34.22.
Forceps, Vacuum, or Other Assisted	Extraction of products of conception, low/mid/high forceps or vacuum or internal version or other, via natural or artificial opening or external approach (ICD Procedure Codes 10D07Z3, 10D07Z4, 10D07Z5, 10D07Z6, 10D07Z7, 10D07Z8, 10E0XZZ).
**Adverse Outcomes**	
In-Hospital Maternal Death	Delivery encounter with discharge status "Expired".
Pre-existing Chronic Hypertension	Delivery encounter with ICD 10 Diagnosis Code containing "O10" (Pre-existing hypertension complicating pregnancy, childbirth and the puerperium).
Gestational Hypertension	Delivery encounter with ICD 10 Diagnosis Code containing "O13" (Gestational [pregnancy-induced] hypertension without significant proteinuria) or "O16" (Unspecified maternal hypertension).
Pre-eclampsia (mild,severe)	Delivery encounter with ICD 10 Diagnosis Code containing "O14" (Pre-eclampsia).
Eclampsia	Delivery encounter with ICD 10 Diagnosis Code containing "O15" (Eclampsia).
Chronic Hypertension with Superimposed Pre-eclampsia	Delivery encounter with ICD 10 Diagnosis Code containing "O11" (Pre-existing hypertension with pre-eclampsia).
Air and Thrombotic Embolism	Delivery encounter with ICD 10 Diagnosis Code as defined by CDC SMM.
Sepsis	Delivery encounter with ICD 10 Diagnosis Code as defined by CDC SMM.
Ante-, Intra-, or Post-partum Hemorrhage	Delivery encounter with ICD 10 Diagnosis Code containing "O46" (Antepartem), "O67" (Intrapartem) or "072" (Postpartem).
Acute Myocardial Infarction	Delivery encounter with ICD 10 Diagnosis Code containing "I21" (AMI) or "I22" (STEMI/NSTEMI).
Cardiomyopathy	Delivery encounter with ICD 10 Diagnosis Code containing "I21" (Cardiomyopathy) or "I22" (Cardiomyopathy in diseases classified elsewhere
COVID-19	Delivery encounter with ICD 10 Diagnosis Code containing "U07.1" (COVID-19) or "U09" (Post-COVID Condition).

© 2022 Molina RL et al. *JAMA Network Open.*

eTable 3. Elixhauser Comorbidities, During COVID-19 vs Pre–COVID-19

	Pre-COVID Jan 2019-Feb 2020	Post-COVID Mar 2020-Apr 2021	Standardized Mean Difference
**Acquired immune deficiency syndrome**	567 (0.1%)	621 (0.1%)	0.004
**Alcohol abuse**	1024 (0.1%)	1079 (0.1%)	0.004
**Deficiency anemias**	72922 (8.6%)	79743 (9.9%)	0.046
**Arthropathies**	3201 (0.4%)	3371 (0.4%)	0.007
**Chronic blood loss**	82953 (9.8%)	87615 (10.9%)	0.037
**Cancer, leukemia**	87 (0%)	105 (0%)	0.003
**Cancer, lymphoma**	61 (0%)	78 (0%)	0.003
**Cancer, metastatic**	44 (0%)	38 (0%)	-0.001
**Cancer, in situ**	176 (0%)	163 (0%)	0.000
**Cancer, malignant**	243 (0%)	218 (0%)	-0.001
**Cerebrovascular disease**	131 (0%)	128 (0%)	0.000
**Congestive heart failure**	282 (0%)	323 (0%)	0.004
**Coagulopathy**	18716 (2.2%)	20111 (2.5%)	0.019
**Dementia**	12 (0%)	11 (0%)	0.000
**Depression**	32999 (3.9%)	38311 (4.8%)	0.043
**Diabetes, chronic**	2626 (0.3%)	2740 (0.3%)	0.005
**Diabetes, no chronic conditions**	46657 (5.5%)	51340 (6.4%)	0.037
**Drug abuse**	22451 (2.6%)	23514 (2.9%)	0.017
**Hypertension, complicated**	16813 (2%)	17723 (2.2%)	0.015
**Hypertension, uncomplicated**	4856 (0.6%)	5105 (0.6%)	0.008
**Liver disease, mild**	6355 (0.7%)	6168 (0.8%)	0.002
**Liver disease, severe**	64 (0%)	67 (0%)	0.001
**Chronic pulmonary disease**	45720 (5.4%)	49389 (6.1%)	0.032
**Neurological disorders affecting movement**	336 (0%)	388 (0%)	0.004
**Other neurological disorders**	13208 (1.6%)	14062 (1.7%)	0.015
**Seizures and epilepsy**	4290 (0.5%)	4383 (0.5%)	0.005
**Obesity**	107869 (12.7%)	120374 (14.9%)	0.065
**Paralysis**	383 (0%)	384 (0%)	0.001
**Peripheral vascular disease**	311 (0%)	345 (0%)	0.003
**Psychoses**	8747 (1%)	9471 (1.2%)	0.014
**Pulmonary circulation disease**	191 (0%)	183 (0%)	0.000
**Renal failure, moderate**	673 (0.1%)	695 (0.1%)	0.002
**Renal failure, severe**	118 (0%)	115 (0%)	0.000
**Hypothyroidism**	32521 (3.8%)	32420 (4%)	0.010
**Other thyroid disorders**	5541 (0.7%)	5949 (0.7%)	0.010
**Peptic ulcer with bleeding**	90 (0%)	85 (0%)	0.000
**Valvular disease**	1593 (0.2%)	1618 (0.2%)	0.003
**Weight loss**	124 (0%)	105 (0%)	-0.001

© 2022 Molina RL et al. *JAMA Network Open.*

eTable 4. Sensitivity Analysis: Multivariate Linear Regression for Absolute Difference in Length of Stay by Mode of Delivery, During COVID-19 vs Pre–COVID-19

	**Difference During COVID vs Pre-COVID**	**P-value**
**Length of Stay**		
Length of Stay-All Live Births, mean	-0.183 (-0.19, -0.176)	<0.0001
Length of Stay-Vaginal, mean	-0.133 (-0.14, -0.127)	<0.0001
Length of Stay-Cesarean, mean	-0.294 (-0.309, -0.279)	<0.0001
Length of Stay-Assisted, mean	-0.132 (-0.153, -0.111)	<0.0001

© 2022 Molina RL et al. *JAMA Network Open.*

eTable 5. Sensitivity Analysis: Relative Difference in Obstetric Outcomes, Pregnancies That Took Place Fully During the Pandemic vs Those Without Exposure to the Pandemic

	Odds Ratio	P-value
**Mortality Outcomes**		
Fetal Deaths/Stillbirths	1.01 (0.94, 1.08)	0.811
Maternal Deaths During Delivery Hospitalization	1.8 (0.86, 3.75)	0.116
**Mode of Delivery**		
Vaginal	1.03 (1.01, 1.04)	<0.001
Vaginal Birth After Cesarean	0.98 (0.94, 1.03)	0.414
Primary Cesarean	1 (0.98, 1.02)	0.838
Repeat Cesarean	0.96 (0.94, 0.98)	<0.001
Forceps, Vacuum, and Other Assisted	0.98 (0.94, 1.02)	0.344
**Complications**		
Pre-existing Chronic Hypertension	1.06 (1.01, 1.1)	0.008
Chronic Hypertension with Superimposed Pre-eclampsia	0.98 (0.92, 1.04)	<0.001
Gestational Hypertension	1.13 (1.09, 1.17)	<0.001
Pre-eclampsia (mild, severe)	1.06 (1.03, 1.09)	0.826
Eclampsia	1.02 (0.84, 1.24)	0.425
Sepsis	0.84 (0.72, 0.99)	0.542
Obstetric Hemorrhage	1.093 (1.05, 1.13)	0.035
Acute Myocardial Infarction	1.09 (0.56, 2.13)	<0.001
Cardiomyopathy	1.15 (0.85, 1.56)	0.804
Venous Thromboembolism Events	1.09 (0.83, 1.43)	0.364
	**Rate Ratio**	**P-value**
**Length of Stay**		
Length of Stay-All Live Births, mean	-0.16 (-0.172, -0.148)	<0.0001
Length of Stay-Vaginal, mean	-0.106 (-0.118, -0.094)	<0.0001
Length of Stay-Cesarean, mean	-0.269 (-0.297, -0.241)	<0.0001
Length of Stay-Assisted, mean	-0.117 (-0.156, -0.077)	<0.0001

© 2022 Molina RL et al. *JAMA Network Open.*

eTable 6. Unadjusted Obstetric Outcomes, During COVID-19 vs Pre–COVID-19

Outcome	Pre-COVID Jan 2019-Feb 2020	COVID Mar 2020-Apr 2021
**Live Birth Outcomes**		
Pre-term	92140 (10.7%)	86829 (10.7%)
Term	766892 (89.3%)	726951 (89.3%)
**Mortality Outcomes**		
In-hospital Maternal Death	44 (0.01%)	70 (0.1%)
Maternal Death with COVID-19 Diagnosis	0 (0.0%)	1 (0.0%)
Fetal Death/Stillbirth	7469 (0.9%)	7196 (0.9%)
**Mode of Delivery**		
Forceps, Vacuum or Other Assisted	31637 (3.7%)	30053 (3.7%)
Primary Cesarean	147672 (17.0%)	144107 (17.6%)
Repeat Cesarean	134857 (15.6%)	127326 (15.5%)
Vaginal	532509 (61.5%)	501273 (61.1%)
VBAC	19826 (2.3%)	18217 (2.2%)
**Length of Stay**		
Length of Stay-All live births	2.66	2.49
Length of Stay-Vaginal	2.26	2.13
Length of Stay-Cesarean	3.46	3.18
Length of Stay-Assisted	2.52	2.39
**Complications**		
Pre-existing Chronic Hypertension	27177 (3.2%)	29240 (3.6%)
Chronic Hypertension with Superimposed Pre-eclampsia	9883 (1.2%)	10209 (1.3%)
Gestational Hypertension	75841 (8.9%)	79333 (9.9%)
Pre-eclampsia (mild, severe)	53479 (6.3%)	53724 (6.7%)
Eclampsia	808 (0.1%)	806 (0.1%)
Sepsis	882 (0.1%)	768 (0.1%)
Ante-, Intra-, or Post-partum Hemorrhage	43401 (5.1%)	44120 (5.5%)
Acute Myocardial Infarction	55 (0%)	72 (0%)
Cardiomyopathy	393 (0%)	392 (0%)
Venous Thromboembolism Events	456 (0.1%)	453 (0.1%)

© 2022 Molina RL et al. *JAMA Network Open.*

eTable 7. Multivariate Logistic Regression for Relative Difference in Obstetric Outcomes Across Race and Ethnicity, During COVID-19 vs Pre–COVID-19

	OR Black v White	OR Hispanic v White	OR Asian v White	OR Other v White
**Mortality Outcomes**				
Fetal Death/Stillbirth	0.986 (0.902, 1.078)	1.009 (0.908, 1.122)	0.904 (0.692, 1.182)	0.979 (0.876, 1.095)
In-Hospital Maternal Death	0.465 (0.172, 1.256)	1.127 (0.397, 3.199)	0.968 (0.144, 6.487)	0.346 (0.083, 1.444)
**Mode of Delivery**				
Vaginal	1.007 (0.986, 1.029)	1.009 (0.99, 1.028)	1.02 (0.987, 1.053)	1.012 (0.987, 1.038)
Vaginal Birth After Cesarean	1.035 (0.979, 1.095)	1.026 (0.964, 1.091)	1.024 (0.934, 1.122)	1.065 (0.991, 1.144)
Primary Cesarean	0.978 (0.951, 1.006)	1.003 (0.972, 1.034)	0.966 (0.925, 1.008)	0.994 (0.96, 1.029)
Repeat Cesarean	0.994 (0.969, 1.019)	0.99 (0.964, 1.016)	1.023 (0.979, 1.069)	0.987 (0.954, 1.021)
Forcep, Vacuum, and Other Assisted	1.023 (0.969, 1.08)	1.008 (0.957, 1.062)	0.963 (0.902, 1.029)	0.947 (0.891, 1.006)
**Length of Stay**				
Length of Stay-All Live Births, mean	1 (0.993, 1.008)	**1.016 (1.007, 1.024)**	0.994 (0.983, 1.006)	0.993 (0.985, 1.002)
Length of Stay-Vaginal, mean	1 (0.991, 1.009)	**1.021 (1.01, 1.033)**	0.993 (0.982, 1.004)	1.002 (0.994, 1.011)
Length of Stay-Cesarean, mean	1.003 (0.99, 1.017)	1.007 (0.995, 1.019)	0.993 (0.971, 1.016)	**0.981 (0.966, 0.996)**
Length of Stay-Assisted, mean	1.005 (0.982, 1.029)	1.003 (0.982, 1.025)	1.027 (0.99, 1.066)	1.011 (0.986, 1.037)
**Complications**				
Pre-existing Chronic Hypertension	**0.949 (0.904, 0.996)**	1.056 (0.993, 1.122)	1.051 (0.926, 1.193)	0.952 (0.886, 1.023)
Chronic Hypertension with Superimposed Pre-eclampsia	0.982 (0.911, 1.058)	0.987 (0.891, 1.095)	0.998 (0.826, 1.206)	1.005 (0.944, 1.069)
Gestational Hypertension	1.007 (0.97, 1.046)	0.986 (0.944, 1.031)	1 (0.934, 1.07)	1.011 (0.95, 1.074)
Pre-eclampsia (mild, severe)	0.99 (0.949, 1.032)	0.977 (0.938, 1.018)	1.056 (0.974, 1.145)	1.002 (0.646, 1.554)
Eclampsia	0.851 (0.652, 1.111)	1.126 (0.825, 1.539)	0.572 (0.2, 1.638)	1.027 (0.924, 1.142)
Sepsis	0.976 (0.683, 1.396)	0.928 (0.646, 1.334)	1.241 (0.726, 2.121)	1.169 (0.628, 2.175)
Obstetric Hemorrhage	0.983 (0.93, 1.038)	1.002 (0.952, 1.055)	0.977 (0.91, 1.048)	0.88 (0.584, 1.326)
Acute Myocardial Infarction	0.485 (0.147, 1.602)	0.882 (0.173, 4.487)	**-**	1.067 (0.989, 1.151)
Cardiomyopathy	1.15 (0.722, 1.832)	0.907 (0.541, 1.52)	1.565 (0.451, 5.434)	1.293 (0.189, 8.865)
Venous Thromboembolism Events	0.965 (0.597, 1.558)	1.216 (0.754, 1.96)	0.879 (0.29, 2.66)	0.729 (0.386, 1.377)

Legend:
Bolded values are statistically significant with p-values < 0.05.

© 2022 Molina RL et al. *JAMA Network Open.*

eFigure 1. Comparing Live Birth Data From US Census and PHD, 2021 and 2020 vs 2019

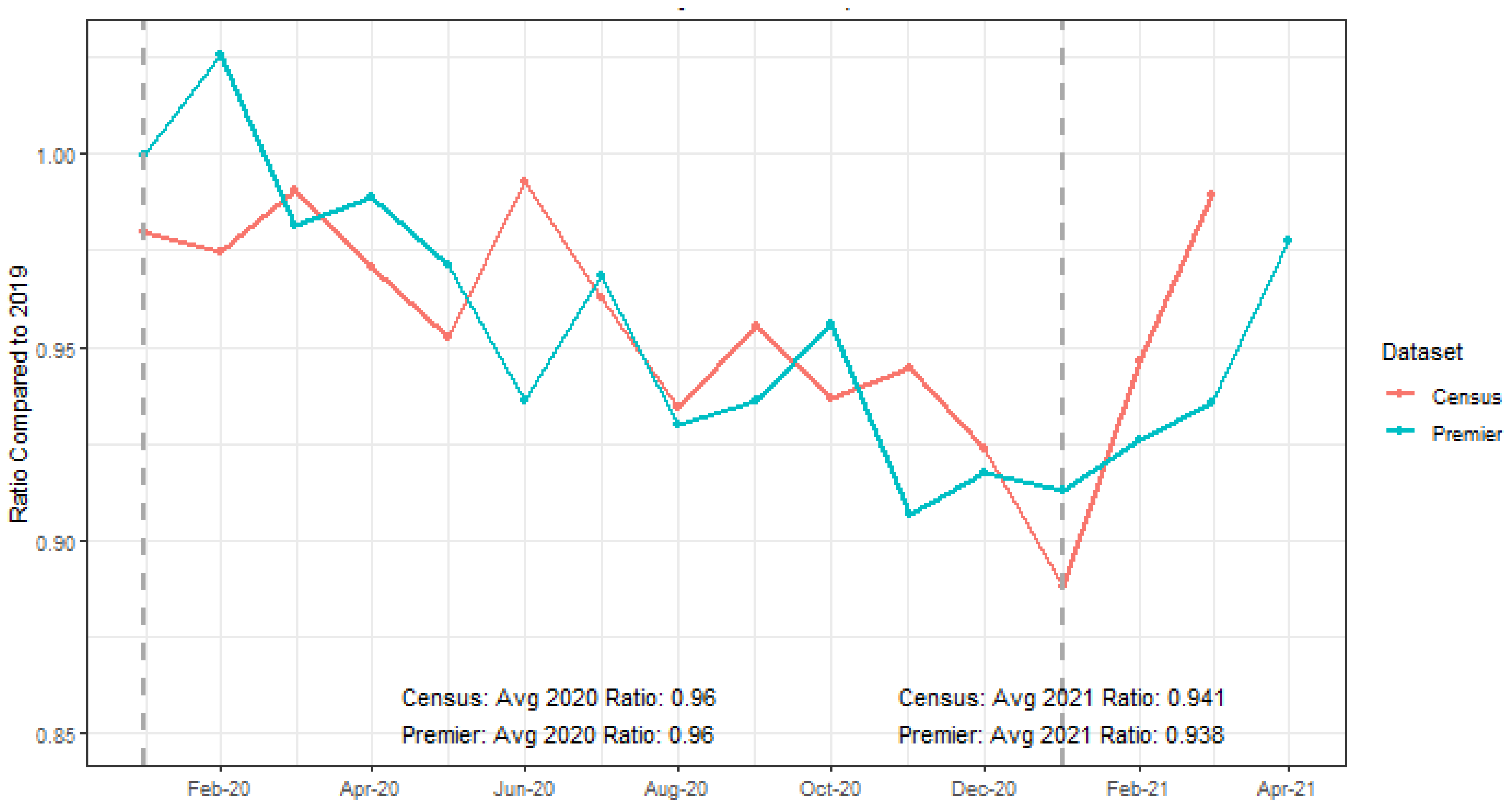

© 2022 Molina RL et al. *JAMA Network Open.*

eFigure 2. Rates of Complications During Pregnancy and Birth by Race and Ethnicity, January 2019 to April 2021

a) Gestational Hypertension

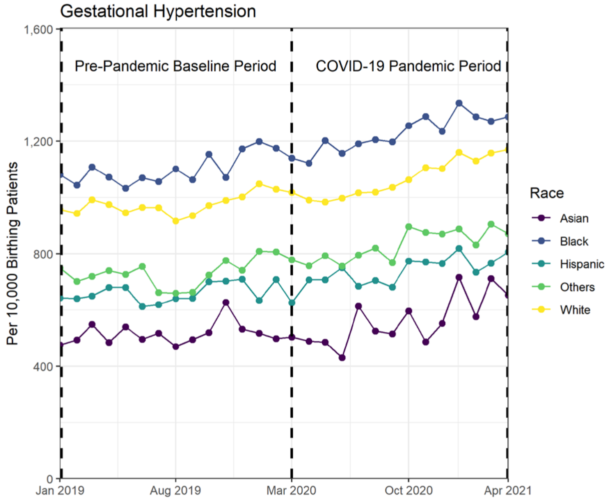

b) Pre-eclampsia (mild, severe)

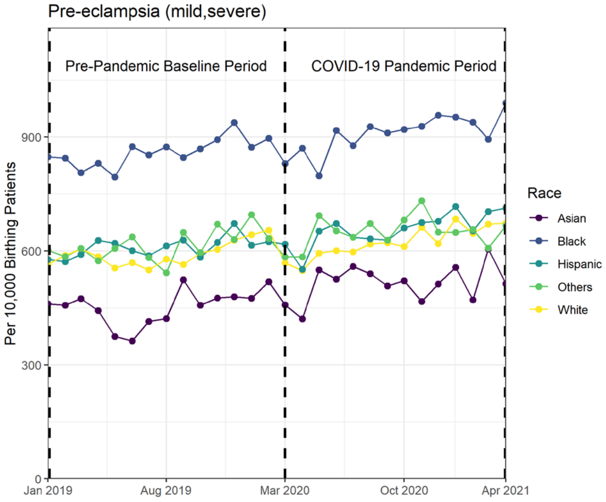

c) Eclampsia

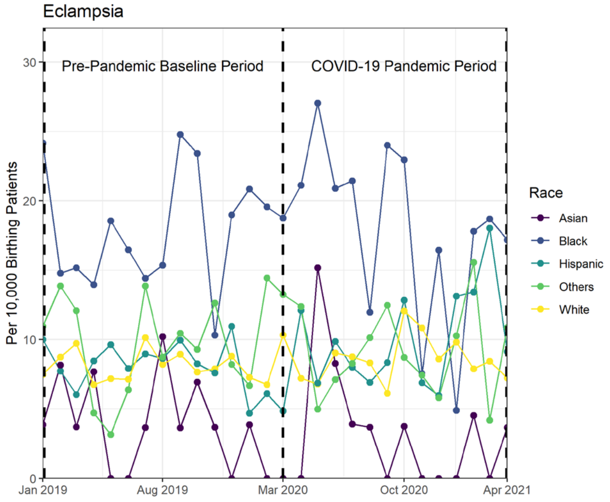

d) Pre-existing Chronic Hypertension

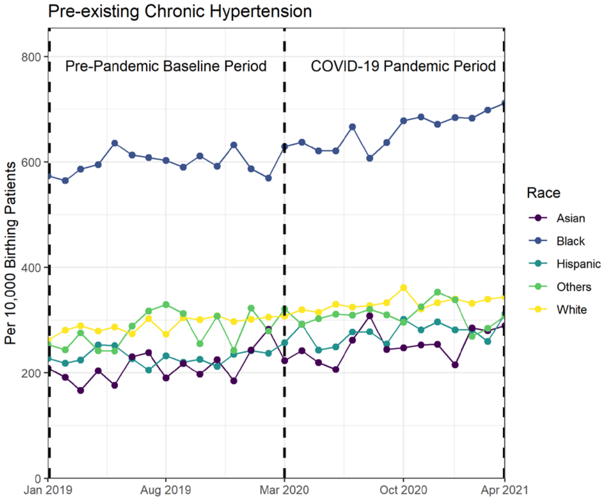

© 2022 Molina RL et al. *JAMA Network Open.*

e) Chronic Hypertension with Superimposed Pre-eclampsia

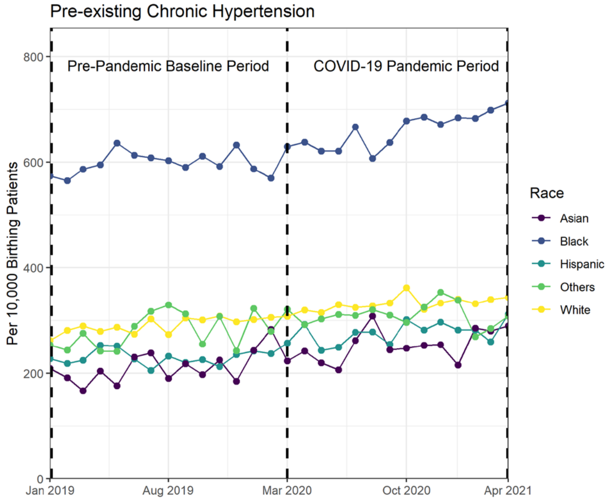

f) Acute Myocardial Infarction

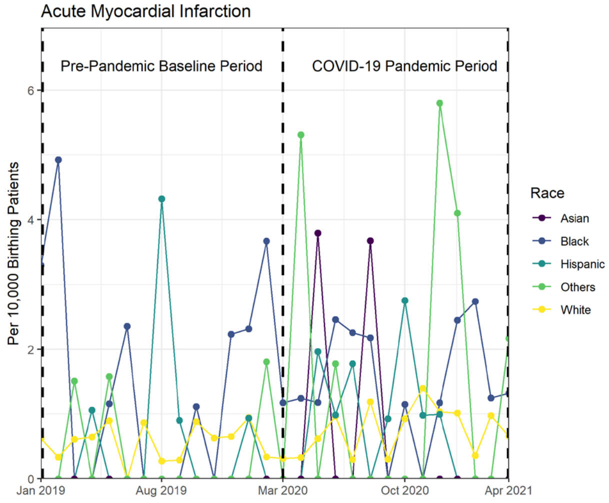

g) Cardiomyopathy

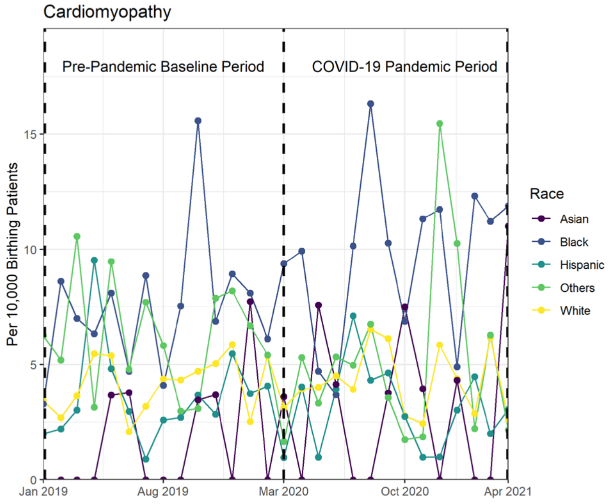

h) Venous Thromboembolism Event

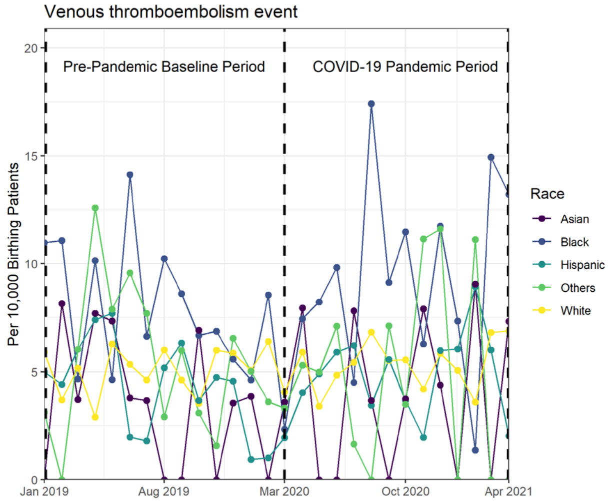

© 2022 Molina RL et al. *JAMA Network Open.*

i) Obstetric Hemorrhage

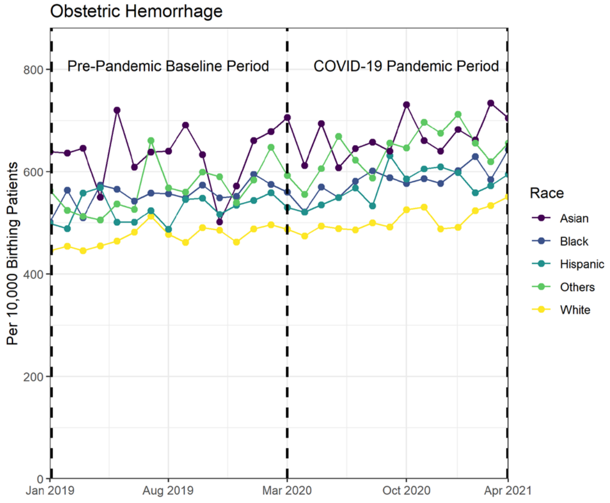

j) Sepsis

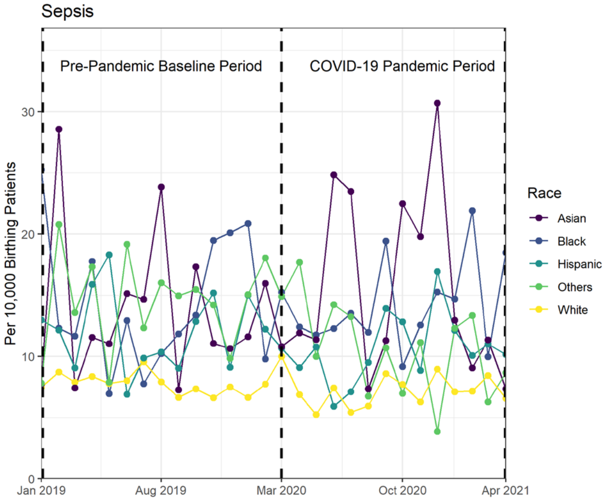

© 2022 Molina RL et al. *JAMA Network Open.*
